# Special Issue “Molecular Dynamics Simulations and Structural Analysis of Protein Domains (2nd Edition)”

**DOI:** 10.3390/ijms27146472

**Published:** 2026-07-21

**Authors:** Alexandre G. de Brevern

**Affiliations:** 1 Université Paris Cité and Université de la Réunion, INSERM, EFS, BIGR U1134, DSIMB Bioinformatics Team, 75015 Paris, France; alexandre.debrevern@univ-paris-diderot.fr; 2 Université Paris Cité and Université de la Réunion, INSERM, EFS, BIGR U1134, DSIMB Bioinformatics Team, 97744 Saint Denis, France; 3 Initiatives IdEx Globule Rouge d’Excellence (InIdex GR-Ex), Université Paris Cité, 75015 Paris, France

Three-dimensional (3D) protein structures provide the essential framework for understanding molecular function. However, these structures offer only static snapshots of systems whose function is inherently dynamic. Over the past decade, structure prediction has undergone a substantial transformation through the utilisation of deep learning methodologies, notably AlphaFold2, and through the rapid augmentation of publicly accessible structural models [1,2,3]. This advancement has led to a significant enhancement in structural coverage; however, it has not yet addressed the necessity of characterising conformational heterogeneity. Protein regions can be classified as rigid, flexible, highly flexible, or intrinsically disordered, with these properties exhibiting variability in response to the molecular environment, post-translational modifications, ligand binding, and interactions with partner molecules [4,5,6]. Consequently, a single structure is capable of describing only a single point, or at best a single basin, within a broader conformational landscape.

Molecular dynamics (MD) simulations provide a direct computational route for exploring these landscapes at atomic resolution. Classical MD, enhanced-sampling methods, and related free-energy approaches have been shown to reveal changes in local geometry, interaction networks, hydration, domain organisation, and collective motions that are inaccessible from static coordinates alone [7,8,9]. This information is of particular value in the study of allostery, disease-associated variants, phosphorylation, ligand inhibition, substrate selectivity, and transient binding pockets. However, it is important to note that an MD trajectory does not constitute an experimental observation and cannot be interpreted as a complete equilibrium ensemble. The mechanistic value of the approach is contingent upon the quality of the starting structure, the force field, the simulated environment, the sampling strategy, the use of independent replicates, and the convergence of the quantities being analysed [10,11]. These considerations are pivotal to the interpretation of the studies assembled in this Special Issue.

The initial edition of the Special Issue, bearing the title “Molecular Dynamics Simulations and Structural Analysis of Protein Domains”, demonstrated the extensive range of structural modelling and simulation methodologies employed in the context of proteins of biomedical significance [12]. The present second edition continues this perspective through eight original contributions. The topics covered include engineered multidomain proteins, kinase inhibition, coronavirus-receptor recognition, cryptic-site prediction, chaperone-associated structuralisation, sequence-structure motifs, and enzyme substrate selection. The collection also reflects a methodological evolution, whereby atomistic simulations are increasingly combined with nuclear magnetic resonance (NMR) spectroscopy, structural alphabets [13], enhanced sampling, Markov state models, statistical analyses, machine learning, and high-performance computing. The articles demonstrate that protein dynamics should not be considered a separate layer that is added after structure determination, but rather an integral component of structural interpretation. Figure 1 provides a synopsis of the methodological organisation, illustrating the progression of the eight contributions from hybrid structural characterisation and classical MD to enhanced sampling, data-driven discovery, and theory- or bioinformatics-based structural analysis.

The initial contribution, by Konshina et al. [14], pertains to two HER2-targeting fusion proteins that have been constructed from the Designed ankyrin repeat protein (DARPin) G3 module and an albumin-binding domain. DARPins are a class of compact, non-immunoglobulin scaffold proteins that exhibit favourable stability and binding properties. However, their small size can result in rapid renal clearance. The fusion of an albumin-binding module has been shown to increase circulatory residence time. However, it should be noted that the resulting multidomain construct cannot be assumed to behave as the simple sum of its isolated components [22]. The authors elegantly compared G3-ABD and ABD-G3 chimaeras using a range of biophysical methods, including nuclear magnetic resonance spectroscopy, homology modelling, docking, Monte Carlo sampling, and atomistic MD simulations. It was evident that both constructs formed extensive interdomain contacts; however, they differed in their preferred interfaces and conformational exchange. It is further postulated that a nonpolar surface of G3, which is involved in HER2 recognition, could also participate in intramolecular association with ABD. The flexible linker has been shown to constrain the relative accessibility of the interacting surfaces. HSA binding to ABD shifted the interdomain equilibrium by competing with G3 for the ABD interaction surface. The present study provides a clear example of how domain order, linker geometry, and transient interdomain packing can determine the functional behaviour of engineered protein therapeutics.

Vu et al. [15] investigated the effect of Ruxolitinib on the Janus homology 1 (JH1) kinase domain of Janus kinase 2 (JAK2), an essential mediator of cytokine signalling. Deregulated JAK2 activity is associated with myeloproliferative neoplasms, and the JAK2 V617F mutation has been identified as a significant molecular event in polycythaemia vera and a substantial fraction of essential thrombocythemia and primary myelofibrosis cases [23]. The study made use of four JH1 systems, each representing a different phosphorylation and inhibitor-binding state. Conventionally, fluctuations in protein conformation are measured. However, in addition to these measurements, the local conformational behaviour of the protein was analysed using Protein Blocks and the associated *N*_eq_ index [13]. The *N*_eq_ index is a quantitative measure of the diversity of backbone conformations sampled at each position [24]. The simulations indicated that phosphorylation modified the mobility of the C-helix and neighbouring functional regions. In addition, in the mono-phosphorylated system, Ruxolitinib reduced flexibility in several functional regions and shifted the ensemble towards an inactive-like dynamic state, whereas extensive phosphorylation partly counteracted this stabilisation. The results obtained lend support to a hypothesis in which inhibition is not limited to occupancy of the ATP-binding site, but also involves restriction of the conformational plasticity required for activation. Furthermore, it is demonstrated that local structural alphabets can complement RMSD and RMSF by distinguishing local backbone rearrangements from larger-scale displacements.

In their study, Tang et al. [16] examined the ongoing evolution of SARS-CoV-2 by comparing complexes formed between human ACE2 and receptor-binding domains from the ancestral virus, the Delta variant, and multiple Omicron subvariants. The experimental structure of the hACE2-RBD complex provides a robust structural foundation for such comparisons [25], but the accumulation of mutations changes electrostatic complementarity, hydrogen-bond networks, local flexibility, and the distribution of accessible conformational states. The authors combined MD simulations with MM/GBSA calculations and potential-of-mean-force profiles. The analyses conducted did not reveal a monotonic increase in hACE2 affinity during viral evolution.

Delta and BA.1 were consistently classified as strong binders by both MM/GBSA and potential-of-mean-force calculations, whereas later Omicron subvariants displayed less uniform energetic profiles. In particular, XBB.1.16 yielded method-dependent affinity estimates, XBB.1.9.1 was predicted to bind less strongly than BA.1, and JN.1 sampled two principal conformational populations corresponding to higher- and lower-affinity states. This bimodal behaviour provides a plausible mechanistic hypothesis for balancing receptor engagement and conformational adaptability, with possible implications for immune evasion. This observation indicates that evolutionary success cannot be reduced to the maximisation of receptor binding alone and may instead reflect trade-offs among receptor engagement, conformational adaptability, and immune escape. The work also illustrates why conclusions based on one energy estimator should be treated with caution and why agreement among structural, interaction, and free-energy descriptors is more informative than any isolated score.

Motono et al. [18] introduced CrypTothML, a framework designed to identify cryptic binding sites by combining mixed-solvent MD with supervised machine learning. Cryptic sites are transient or inducible pockets that may not be apparent in an apo experimental structure but can become accessible through conformational changes, rendering them attractive targets for allosteric drug discovery [26]. Six probe molecules of differing chemical composition were utilised in mixed-solvent simulations to identify surface hotspots. Features describing probe occupancy and the physicochemical and geometrical properties of the surrounding protein surface were then used to distinguish cryptic from non-cryptic hotspots. On the curated dataset utilised in the study, the selected AdaBoost model attained a ROC AUC of approximately 0.88 and a precision-recall AUC of approximately 0.80, thereby demonstrating superior performance in comparison to the comparator methods evaluated by the authors. Within the benchmark used by the authors, CrypTothML outperformed the compared machine-learning methods and provided an effective integration of MSMD-derived and protein-surface features. A notable finding was that complete pocket opening was not always necessary during the simulations, as subtle alterations in surface shape, charge density, hydrophobicity, and probe accumulation already provided predictive insights. The method provides a productive illustration of physics-informed machine learning; however, its generalisation will be contingent on the operational definition of a cryptic site, the size and diversity of the training data, and prospective validation on targets that were not represented during model development.

In the study by Roterman et al. [20], the interaction of casein-derived polypeptide segments with the Hsp104 chaperone and a disaggregase was examined using the fuzzy oil drop model. Heat-shock proteins and molecular chaperones have been shown to assist proteins in evading non-native interactions and aggregation, particularly under conditions of cellular stress [27]. The fuzzy oil drop approach is a method of evaluating the distribution of hydrophobicity within a protein structure relative to an idealised micelle-like organisation expected in an aqueous environment. In this analysis, the chaperone assembly is interpreted as an external force field that can stabilise a conformation different from that preferred by an isolated polypeptide in water. The Hsp104 complex exhibited a hydrophobicity distribution that was significantly different from the idealised centric core, while its constituent pseudo-domains were found to be individually more compatible with aqueous folding. The casein segment was maintained in an extended state within this environment, and the disaggregase was interpreted as preventing inappropriate association. Within the assumptions of the fuzzy oil drop model, the chaperone and disaggregase assemblies were interpreted as external force fields that stabilise casein conformations distinct from those expected for an isolated peptide in water. This contribution is distinct from the atomistic MD studies included in the collection, yet it aligns with the overarching principle that a protein conformation is determined not solely by its sequence, but also by the physical constraints and interaction field imposed by its molecular environment.

Lo et al. [21] provided a complementary large-scale perspective through an analysis of the GXXXG motif, also known as the glycine zipper. This motif is classically associated with close helix–helix packing in membrane proteins, but it also occurs in soluble proteins and structurally heterogeneous regions [28]. By integrating UniProt sequence annotations with available structural information [29], the authors examined more than 25,000 non-redundant protein sequences and more than 72,000 motif occurrences. Motifs were classified according to their transmembrane coverage, and their distributions were assessed using compositional statistics (Shannon entropy, Zipf analysis, power-law modelling, and information-theoretic divergence measures). Transmembrane motifs were less diverse and enriched in hydrophobic residues such as Leucine, Isoleucine, and Valine, whereas non-transmembrane motifs showed greater sequence diversity and more frequent use of small, polar, or flexible residues. A transmembrane coverage threshold of approximately 60% emerged as a useful separator of transmembrane-specific statistical behaviour. These findings demonstrate that the structural meaning of a short sequence motif cannot be inferred independently of its topological and physicochemical context.

Jiang et al. [19] conducted a study on the substrate selection process by poly(A) polymerase, a non-template-dependent nucleotidyltransferase that exhibits a strong preference for the incorporation of ATP over other nucleoside triphosphates. The process of the nucleotide-addition cycle of polymerases necessitates the orchestrated realignment of substrate recognition, active-site organisation, catalysis, and product release [30]. The authors conducted a comparative analysis of apo PAP and complexes with ATP, GTP, CTP, and UTP using Gaussian accelerated MD simulations, docking, MM-PBSA calculations, secondary-structure monitoring, dynamic cross-correlation analysis, and Markov state modelling. ATP binding was associated with the most compact and globally stable ensemble while also producing substrate-specific changes in solvent exposure and local flexibility and induced a loop-to-helix transition around residues 225-230 near the active site. In contrast, the other substrates exhibited a greater propensity for the formation of interdomain openings, a phenomenon that has been demonstrated to facilitate substrate dissociation. The Markov state analysis organised the sampled conformations into metastable conformational states and transition networks, thus obviating the necessity for exclusive reliance on trajectory-averaged observables. The proposed mechanism establishes a correlation between local substrate-specific interactions and larger domain motions, thus providing a framework for the rational engineering of polymerase specificity.

The final contribution, by Kumar et al. [17], focused on the recognition between the MERS-CoV receptor-binding domain and human dipeptidyl peptidase 4, the functional receptor required for viral entry [31]. Three independent microsecond-scale MD simulations were performed under physiological conditions. The analysis identified seven candidate salt-bridge pairs that met the authors’ selection criteria in at least part of the simulations; among them, Asp510-Arg317 and Arg511-Asp393 were the most strongly supported by previous experimental evidence. In addition, five supplementary pairs were put forward for experimental examination. The study provides a focused molecular map of the RBD-DPP4 interface and identifies candidate interactions that could be targeted when designing inhibitors intended to disrupt viral attachment.

A number of recurrent themes are apparent in these contributions. The most fundamental of these is the observation that biological function is encoded by conformational ensembles rather than by a unique structure. This principle is observed in the interdomain organisation of the HER2-targeting chimaeras, the phosphorylation- and inhibitor-dependent behaviour of JAK2 JH1, the variant-dependent hACE2-RBD landscapes, the transient formation of cryptic sites, and the substrate-dependent states of poly(A) polymerase. The studies also demonstrate that local and global dynamics must be considered in unison. Changes to a domain can be confined to a linker, a short loop, a helix, or a contact network. Such changes have been shown to modify domain-scale organisation, alter accessibility to a binding surface, or redistribute the probability of active and inactive states.

A secondary theme is the escalating integration of complementary computational and experimental approaches. NMR spectroscopy is a valuable tool that can provide key constraints on the interpretation of DARPin chimaeras, while structural alphabets can offer a residue-level description of kinase dynamics. Mixed-solvent simulations supply physically motivated features for machine learning, and Markov state models provide a kinetic framework for enhanced-sampling trajectories. Large-scale sequence analyses have been shown to connect structural motifs to evolutionary and topological constraints, while high-performance computing has resulted in increased accessibility of replicate microsecond simulations. Open-source MD software, e.g., Gromacs and NAMD [7,8], in conjunction with community resources including the Protein Data Bank and UniProt [29,32], has rendered these workflows broadly reproducible in principle. In practice, reproducibility still requires complete reporting of starting structures, protonation states, force fields, solvent and ion conditions, equilibration procedures, random seeds, trajectory processing, and analysis thresholds.

The role of artificial intelligence and machine learning should also be defined precisely [33]. In this collection, the most evident methodological integration is provided by CrypTothML, where simulation-derived physical descriptors are used as inputs to a supervised classifier. This hybrid strategy is appealing as it preserves interpretable links between prediction and molecular behaviour. However, it is important to note that performance estimates derived from a curated dataset are not necessarily indicative of transferability to the proteome in its entirety. In principle, the application of machine learning should not be employed to obfuscate uncertainty in the underlying structural data or simulation sampling. Future developments in this field will benefit from external validation sets, prospective benchmarks, uncertainty calibration, and direct comparison with experimentally characterised apo-holo transitions.

Several contributions have potential therapeutic or methodological relevance. The HER2 study informs the design of modular binders, the JAK2 analysis clarifies the dynamic consequences of kinase inhibition, the coronavirus studies identify receptor-interface determinants, and CrypTothML expands the search space for allosteric pockets. The ensuing results can serve as a foundation for the prioritisation of experiments, providing direction for a range of disciplines including mutagenesis, binding assays, medicinal chemistry and protein engineering. Crucially, computational predictions are not substitutes for experimental validation. Predictions of affinity, persistent contacts, cryptic pockets, or allosteric effects are most valuable when they lead to falsifiable hypotheses that can be assessed through NMR, surface plasmon resonance, calorimetry, cryo-EM, kinetic measurements, or cell-based functional assays.

Consequently, significant methodological challenges persist. It is important to note that the length of the simulation is not a sufficient measure of the quality of the sampling. It is possible for a long trajectory to remain confined to a single conformational basin. In order to draw conclusions that are dependent on rare transitions or free-energy differences, it is necessary to employ independent replicates, enhanced-sampling strategies, convergence analyses, and the comparison of alternative estimators. It is evident that force-field limitations and the presence of uncertain protonation or phosphorylation states can assume particular significance at catalytic and protein–protein interfaces. Machine-learning analyses require meticulous management of redundancy and data leakage, while large-scale bioinformatic analyses are contingent on annotation quality and the utilisation of suitable background models. These limitations do not detract from the value of molecular simulation; rather, they delineate the conditions under which its conclusions can be considered reliable.

The eight contributions to this second edition illustrate how protein function emerges from the interplay between structure, conformational variability, molecular environment, and biological context. By combining classical and enhanced molecular dynamics, experimental characterisation, structural modelling, statistical analysis, machine learning, and large-scale bioinformatics, the studies move beyond static structural descriptions towards mechanistic and testable interpretations. Furthermore, it is demonstrated that computational results are strengthened by complementary descriptors, independent sampling, explicit treatment of uncertainty, and comparison with experimental evidence. Across a range of applications, including engineered proteins and enzyme specificity, viral recognition, cryptic-site detection, and sequence motifs, the common objective is to identify molecular determinants that can guide subsequent functional or therapeutic investigations. The future progression of this field will be contingent on the enhancement of reproducible workflows, the integration of heterogeneous data, and the validation of computational hypotheses through experimental methods.

## Figures and Tables

**Figure 1:**
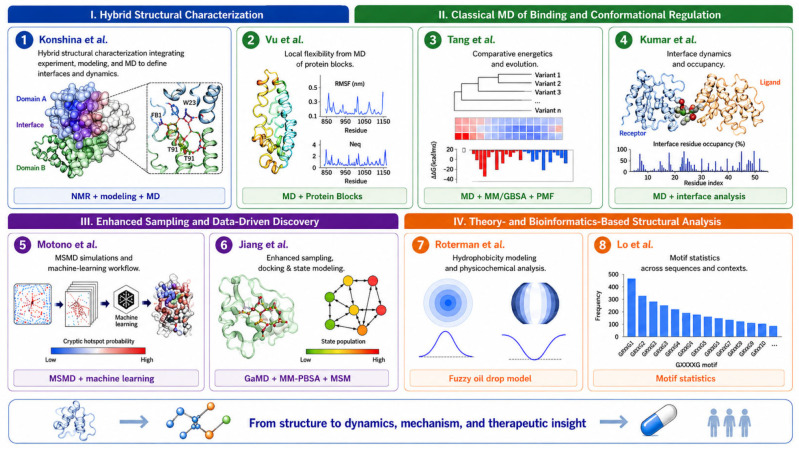
Methodological organisation of the eight contributions included in the Special Issue. The studies are grouped according to four complementary methodological frameworks: hybrid structural characterisation combining experiment, modelling, and MD (Konshina et al.) [14]; classical MD analyses of local conformational variability, comparative binding energetics, and protein–protein interface dynamics (Vu et al. [15], Tang et al. [16], and Kumar et al. [17]); enhanced sampling and data-driven approaches integrating mixed-solvent MD, machine learning, Gaussian accelerated MD, free-energy estimation, and Markov state modelling (Motono et al. [18] and Jiang et al. [19]); and theory- or bioinformatics-based structural analyses using hydrophobicity modelling and large-scale motif statistics (Roterman et al. [20]. and Lo et al. [21]). Together, these approaches connect structural description to conformational dynamics, molecular mechanisms, and potential therapeutic applications.

## Abbreviations

The following abbreviations are used in this manuscript:

ABD	Albumin-binding domain
ACE2	Angiotensin-converting enzyme 2
ATP	Adenosine triphosphate
AUC	Area under the curve
cryo-EM	Cryogenic electron microscopy
CTP	Cytidine triphosphate
DARPin	Designed ankyrin repeat protein
DPP4	Dipeptidyl peptidase 4
GTP	Guanosine triphosphate
hACE2	Human angiotensin-converting enzyme 2
HER2	Human epidermal growth factor receptor 2
HSA	Human serum albumin
Hsp104	Heat shock protein 104
JAK2	Janus Kinase 2
JH1	Janus homology 1
MD	Molecular dynamics
MERS-CoV	Middle East respiratory syndrome coronavirus
MM/GBSA	Molecular mechanics/generalised Born surface area
MM/PBSA	Molecular mechanics/Poisson-Boltzmann surface area
Neq	Equivalent number of Protein Blocks
NMR	Nuclear magnetic resonance
PAP	Poly(A) polymerase
RBD	Receptor-binding domain
RMSD	Root-mean-square deviation
RMSF	Root-mean-square fluctuation
ROC	Receiver operating characteristic
SARS-CoV-2	Severe acute respiratory syndrome coronavirus 2
UTP	Uridine triphosphate
